# Creation and application of war trauma treatment simulation software for first aid on the battlefield based on undeformed high-resolution sectional anatomical image (Chinese Visible Human dataset)

**DOI:** 10.1186/s12909-022-03566-6

**Published:** 2022-06-26

**Authors:** Xin Hu, Li Liu, Zhou Xu, Jingyi Yang, Hongfeng Guo, Ling Zhu, Wouter H. Lamers, Yi Wu

**Affiliations:** 1 Department of Digital Medicine, College of Biomedical Engineering and Medical Imaging, Third Military Medical University (Army Medical University), No. 30, Gaotanyan Street, Shapingba District, 400038 Chongqing, China; 2 Department of Basic Operative Surgery, College of General Medicine, Southwest Hospital, Third Military Medical University (Army Medical University), No. 30, Gaotanyan Street, Shapingba District, 400038 Chongqing, China; 3 Frontier Medical Training Brigade, Third Military Medical University (Army Medical University), No. 75, Dongfeng Street, Hutubi country, 831200 Xinjiang, China; 4 Academic Medical Center, Tytgat Institute for Liver and Intestinal Research, University of Amsterdam, Amsterdam, The Netherlands

**Keywords:** Chinese Visible Human, First aid, War trauma, Sectional anatomy, Self and mutual rescue

## Abstract

**Background:**

Effective first aid on the battlefield is vital to minimize deaths caused by war trauma and improve combat effectiveness. However, it is difficult for junior medical students, which have relatively poor human anatomy knowledge and first aid experience. Therefore, we aim to create a treatment simulation software for war trauma, and to explore its application for first aid training.

**Methods:**

This study is a quantitative post-positivist study using a survey for data collection. First, high-resolution, thin-sectional anatomical images (Chinese Visible Human (CVH) dataset) were used to reconstruct three-dimensional (3D) wound models. Then, the simulation system and the corresponding interactive 3D-PDF, including 3D models, graphic explanation, and teaching videos, were built, and used for first aid training in army medical college. Finally, the interface, war trauma modules, and training effects were evaluated using a five-point Likert scale questionnaire. All measurements are represented as mean and standard deviations. Moreover, free text comments from questionnaires were collected and aggregated.

**Results:**

The simulation software and interactive 3D-PDF were established. This included pressure hemostasis of the vertex, face, head-shoulder, shoulder-arm, upper forearm, lower limb, foot, and punctures of the cricothyroid membrane, pneumothorax, and marrow cavity. Seventy-eight medical students participated in the training and completed the questionnaire, including 66 junior college students and 12 graduate students. The results indicated that they were highly satisfied with the software (score: 4.64 ± 0.56). The systems were user-friendly (score: 4.40 ± 0.61) and easy to operate (score: 4.49 ± 0.68). The 3D models, knowledge of hemostasis, and puncture were accurate (scores: 4.41 ± 0.67, and 4.53 ± 0.69) and easily adopted (scores: 4.54 ± 0.635, and 4.40 ± 0.648). They provided information about hemostasis and puncture (all scores > 4.40), except for cricothyroid membrane puncture (scores: 4.39 ± 0.61), improved the learning enthusiasm of medical students (score: 4.55 ± 0.549), and increased learning interest (score: 4.54 ± 0.57).

**Conclusion:**

Our software can effectively help medical students master first aid skills including hemostasis, cricothyroid membrane and bone marrow puncture, and its anatomy. This may also be used for soldiers and national first aid training.

**Supplementary Information:**

The online version contains supplementary material available at 10.1186/s12909-022-03566-6.

## Background

War trauma is an important cause of combat attrition on the battlefield. According to the casualty data and related reports from the Unites States (US) and United Kingdom (UK) during the wars in Iraq and Afghanistan, vascular injury was a common type of war trauma, with a higher rate than in previous wars [1–3]. E. Glassberg [4], and B.J. Eastridge [5] also reported that death was usually (90%) caused by blood loss. Additionally, airway injury (8%) and tension pneumothorax (1%) were also important major causes of death [4, 6]. However, up to 25% of the deaths were salvageable [5]. Holcomb et al. reported similar rates, of 15% and 19%, among US combat cases [6–8].

Effective first aid on the battlefield, including pressure hemostasis, open airway, and pneumothorax puncture, is an important means to minimize the deaths of combat personnel and improve combat effectiveness. This is vital from the onset of trauma to approximately 10 min after the injury [9]. It is considered the key time of first aid, defined as the’platinum ten minutes’ [10]. During this period, providing correct first aid treatment can significantly shorten the rescue time and improve the rescue rate. However, its effective implementation requires basic knowledge of anatomy, surgery, and military medicine. Soldiers, most of whom are not medical professionals, have relatively poor knowledge of human anatomy, and this makes it difficult for them to accurately judge injuries and implement effective treatment [11]. Current first aid training is mainly conducted on traditional modes, including training mannequins, fake wounds, and animal experiments. They all have shortcomings, including a lack of precise anatomy information and inability to train online, which is limited by time and location [12–16]. It is difficult, therefore, to intuitively acquire detailed human anatomical knowledge about self and mutual rescue in a short period of time.

Constructivism theory is proposed by Swiss psychologist Piaget, which provides a solid theoretical foundation for simulation education in our study. Its core is that learners undertake active learning with the help of necessary learning materials in a certain situation. Studies have reported that the constructivism theory is more suitable for adults because they prefer to learn through experience [17, 18]. It has the potential to facilitate study, communication and cooperation, and has been widely used in many fields, such as medicine, education, trauma resuscitation and entertainment [19–21]. Specially, the three-dimensional (3D) digital human models with detailed human anatomical information are implemented in anatomy teaching and are used for simulated surgery training [22, 23]. Therefore, researchers began to explore its application in the field of war trauma [24].

### Study goal

In this study, it was hypothesized that a simulation software containing relevant first-aid anatomy knowledge had positive effects on mastering first-aid skills quickly. Therefore, we used high-resolution, true-color, thin-sectional anatomical images (Chinese Visible Human datasets, CVH) to build a war trauma treatment simulation software that can truly reflect the Chinese physique. We aim to explore its application in the training for medical students, and apply findings in futures studies and use with soldiers.

## Methods

### Establishment of war trauma treatment software 

**Fig.1:**
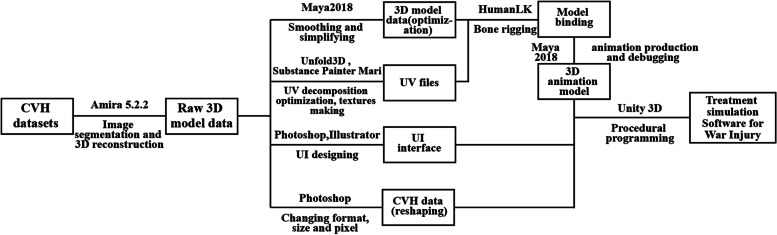
Creation workflow of treatment simulation software on the battlefield. CVH, Chinese Visible Human. 3D, three-dimensional. UI, user interface

CVH datasets have thin-sectional, high-resolution, and true color characteristics and have been widely used in anatomy research and academia [25–28]. In this study, the second CVH dataset (CVH2, image resolution: 3072 × 2048, pixel size: 0.15 mm × 0.15 mm, sectional thickness: 0.5 mm) was used to reconstruct three-dimensional (3D) wound models (http://www.bmicc.cn/web/share/search/cvh). The original CVH2 dataset was segmented to obtain 3D digital models of human muscles, bones, blood vessels, nerves, and other organs using Amira 5.2.2 software (Visage Imaging, Inc., San Diego, California, USA). Then, the post-processing of models, including smoothing, simplifying, texture mapping, and bone rigging were done using Autodesk Maya 2018 (Autodesk, San Rafael, California, USA) and Unfold3d (POLYGONAL DESIGN, Marseille, France). Next, user interface was designed using Photoshop (Adobe, USA) and Illustrator. Finally, Unity3D (Unity Technologies, San Francisco, CA, USA) was used to create the war trauma treatment simulation software. The workflow is shown in Fig. 1.

**Fig.2:**
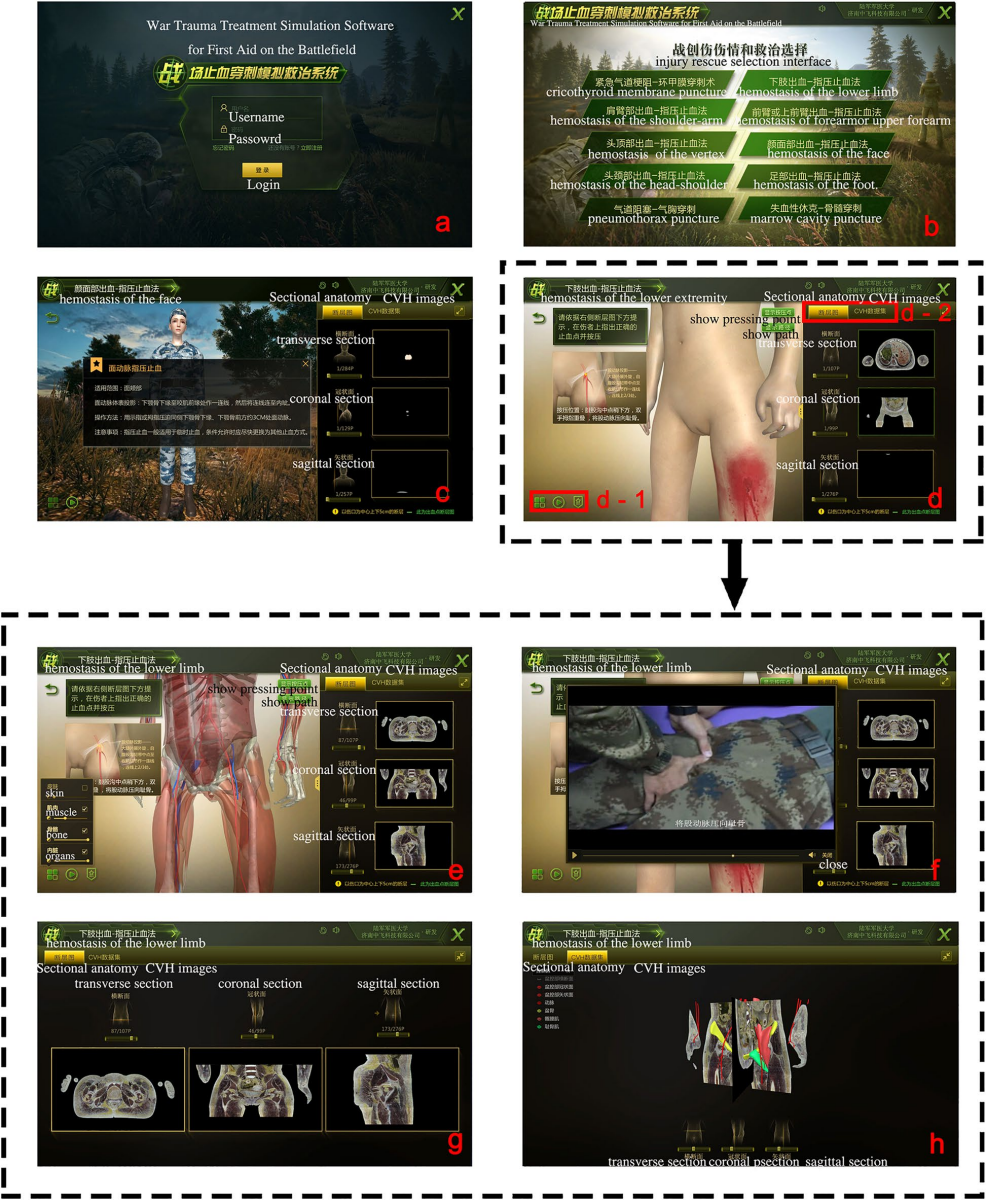
The interfaces of the treatment simulation software a, User login interface; b, Injury treatment module selection interface. c. Basic knowledge of injuries interface; d, Interactive learning interface of the 3D digital anatomy and sectional anatomy; e, Interactive learning interface of the 3D digital anatomy; f, Lecture video interface; g, Interactive learning interface of the sectional anatomy; h, Interactive learning interface of CVH sectional anatomical images and the 3D model; d-1, Clickable area of the 3D model display, and lecture videos; d-2, Sectional anatomical image, CVH and 3D model learning button area

The user interface of the training software was designed as a four-level interface (Fig. 2, Supplementary Fig.S1). The first-level was the login interface, the second-level was the injury treatment module selection interface. The third-level was the basic knowledge of injuries interface, including anatomical and treatment knowledge of the injury. The fourth-level was the interactive learning interface of the 3D digital anatomy and sectional anatomy, which provided a 3D anatomy learning, video course, and sectional anatomy learning of the injury based on the CVH2 dataset.

**Fig.3:**
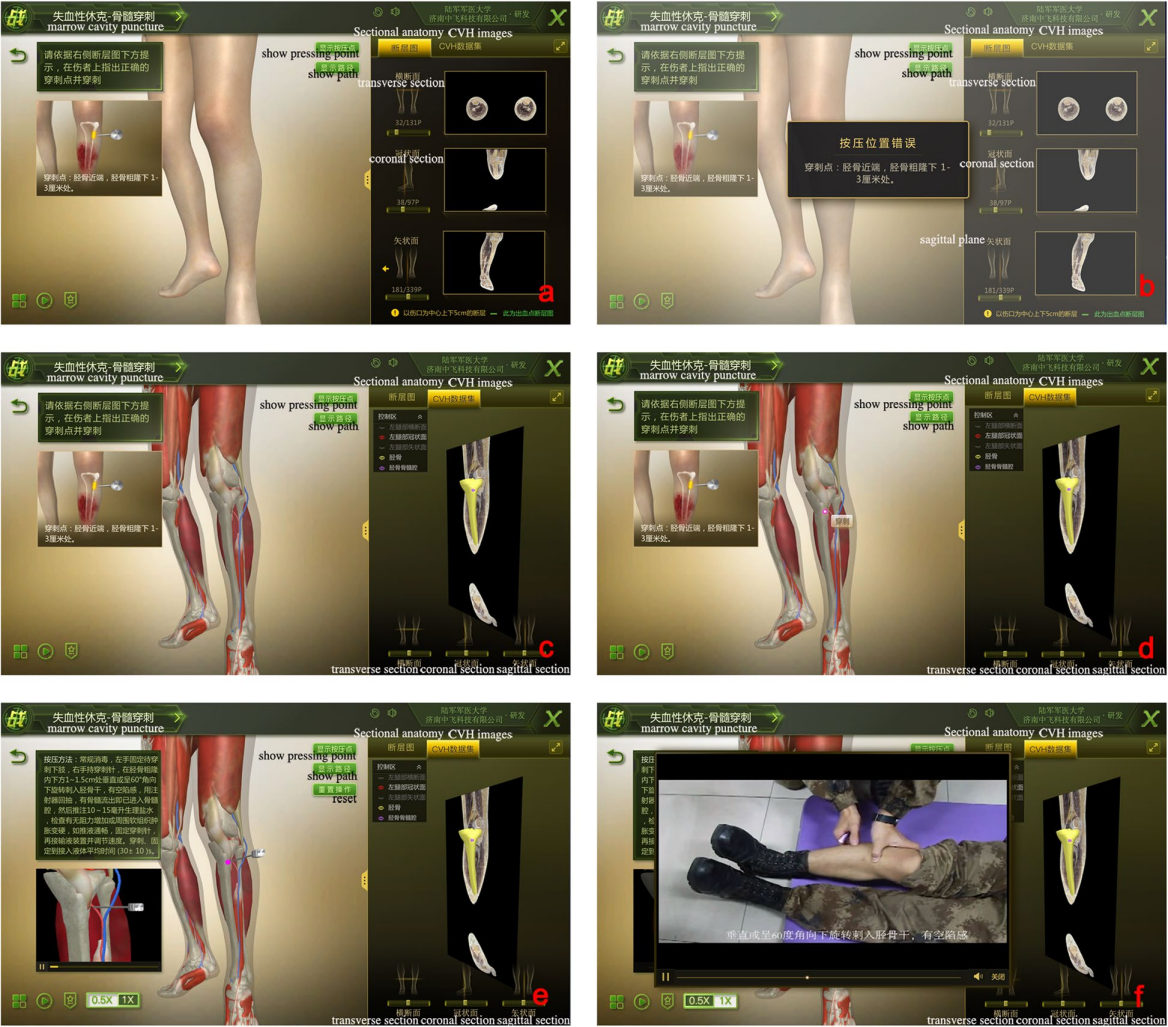
Learning module on bone marrow puncture a, Injury demonstration; b, Error message when selecting the wrong puncture position; c, Demonstration of the correct puncture point of injury after transparency of the skin; d, Selecting of the correct puncture position and confirmation to proceed to the next step; e, Animated demonstration of puncture; f, Teaching video of the correct puncture techniques

In the second-level interface, the injury module included the digital pressure hemostasis and puncture modules. The digital pressure hemostasis modules included the following modules: 1. vertex; 2. face; 3. head-shoulder; 4. the shoulder-arm; 5. upper forearm; 6. lower limb; and 7. foot (Supplementary Fig.S2). The puncture modules included the following parts: 1. cricothyroid membrane; 2. pneumothorax; and 3. marrow cavity (Fig. 3).

In the third-level interface, the software automatically played the injury video, in which the soldier was injured on the battlefield, and explained the trauma characteristics and rescue treatment methods through audio.

In the fourth-level interface, the software demonstrated the compression and puncture point positions and their anatomy. They could zoom, translate, rotate, display, hide, and change the transparency of the digital human body on the left side of the interface, to better observe the 3D anatomy and spatial relationship of the injury. Meanwhile, students can use the sectional anatomy on the right side of the interface. This corresponds to the transverse, coronal, and sagittal sections of the CVH images, at each layer of the 3D model, to better visualize the tomographic anatomy relating to the injury.

If operators want to succeed in the hemostasis modules, they must press the proximal end of the bleeding and press the artery to the bone, rather than the muscle or other structures. If the medical students want to succeed in the puncture modules, they must puncture the correct position. For example, in the marrow cavity puncture module, medical students must puncture the tuberosity of the tibia.

### Creation of an interactive 3D-PDF

**Fig.4:**
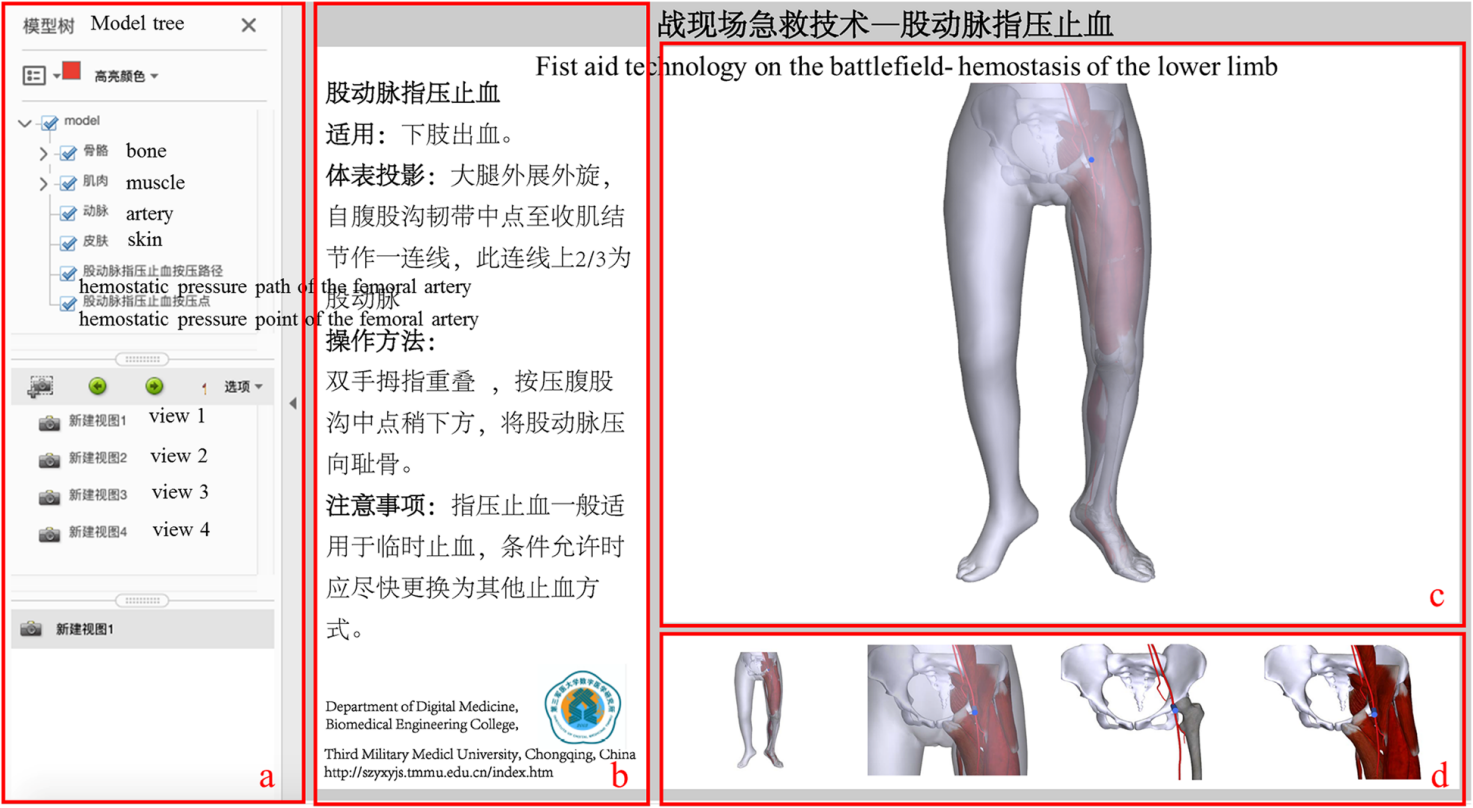
Pressure hemostasis interface of the lower limb in 3D-PDF document a, Functional area of the anatomic model; b, Demonstration area of text of first-aid technology; c, The 3D digital anatomy window; d, Interactive learning interface of the 3D digital anatomy

Using Deep Exploration (Right Hemisphere, Inc, the United States.) and Adobe Acrobat (Adobe Systems Incorporated, San Jose, CA, USA), the interactive 3D-PDF document, including the hemostasis and puncture modules, was created by adding 3D models, graphic interpretation, and teaching videos (Fig. 4). It is a file that supports embedding 3D data and its interactive capabilities. It is used to supplement the simulation software and consolidate the information.

### Design and implementation of the first aid training

The first aid training course included a theoretical course (three class hours) and a practical course (three class hours). In the theoretical part, knowledge of digital pressure hemostasis, cricothyroid membrane puncture, pneumothorax puncture, and marrow cavity puncture and their applied anatomical information were explained to medical students. The simulation software and 3D-PDF were also introduced. During the practical course, medical students were allowed to use the simulation software, and practice the hemostasis and puncture techniques. Before training, the software and 3D-PDF were inserted to the campus network and could be accessed in a browser by downloading the relevant plug-ins.

First aid training on the battlefield was conducted during the digital anatomy and digital medicine introductory classes of academic years 2018–2020 in our school. Excluding the number of repeated elective courses, 80 students participated in the training.

### Evaluation of the software

This study is a quantitative post-positivist study followed by a survey. A questionnaire named “teaching satisfaction questionnaire for the simulation software of hemostasis and puncture on the battlefield”, was designed to evaluate the efficacy of the software from three aspects: software interface, war trauma modules, and training effects. The questionnaire contained 18 items, and the scoring system was based on a five-point Likert scale [29, 30]. Arabic numbers 5, 4, 3, 2, and 1 represented strongly agree, agree, neutral, disagree, and strongly disagree, respectively (Table 2). Army medical students, who participated in the first aid training course, were invited in the evaluation anonymously.

### Statistical analysis

We used mean and standard deviation (Mean ± SD) to describe quantitative variables, and frequency (percent) to describe qualitative variables. Within the group, paired sample t-test was used to compare the results, and independent sample t-test was used to analyze the difference between groups. All statistical tests were performed by SPSS 19, and p < 0.05 was considered significant.

### Ethical statement

This study was approval by the Ethics Committee of the Third Military Medical University (Army Medical University). The experiment was explained to all participants allowing for questions or comments, and informed consent was obtained from all participants. Data and identity were confidential and only used for the purposes of this study. All methods were carried out in accordance with relevant guidelines and regulations.

## Results

Eighty questionnaires were issued to medical students who participated in the training. Seventy-eight valid questionnaires were returned, resulting in a valid questionnaire rate of 97.5%. Sixty-six were junior college students from the troops (Junior college student group: mean age: 21.07 ± 1.50, 23 males) (Table 1), majoring in combat rescue and nursing. Twelve were graduate students (Graduate student group: mean age: 23.92 ± 1.11, 8 males), majoring in Clinical Medicine, Medical Imaging and Nuclear Medicine. All students are in the second semester of the first year of college, and all junior college students just finished the course on system anatomy, but did not attend the course on sectional anatomy. The Cronbach’s alpha value was 0.882, which shows that these questions are reliable.

**Table 1:** Demographic characteristics of all students

Characteristic	Total (*n* = 78)	Junior college student group (*n* = 66)	Graduate student group (*n* = 12)
Age, years	21.5 ± 1.77	21.07 ± 1.50	23.92 ± 1.11
Sex (%)			
Male	31(39.7%)	23 (34.8%)	8 (66.7%)
Female	47(60.3%)	43 (65.2%)	4 (33.3%)

**Fig. 5:**
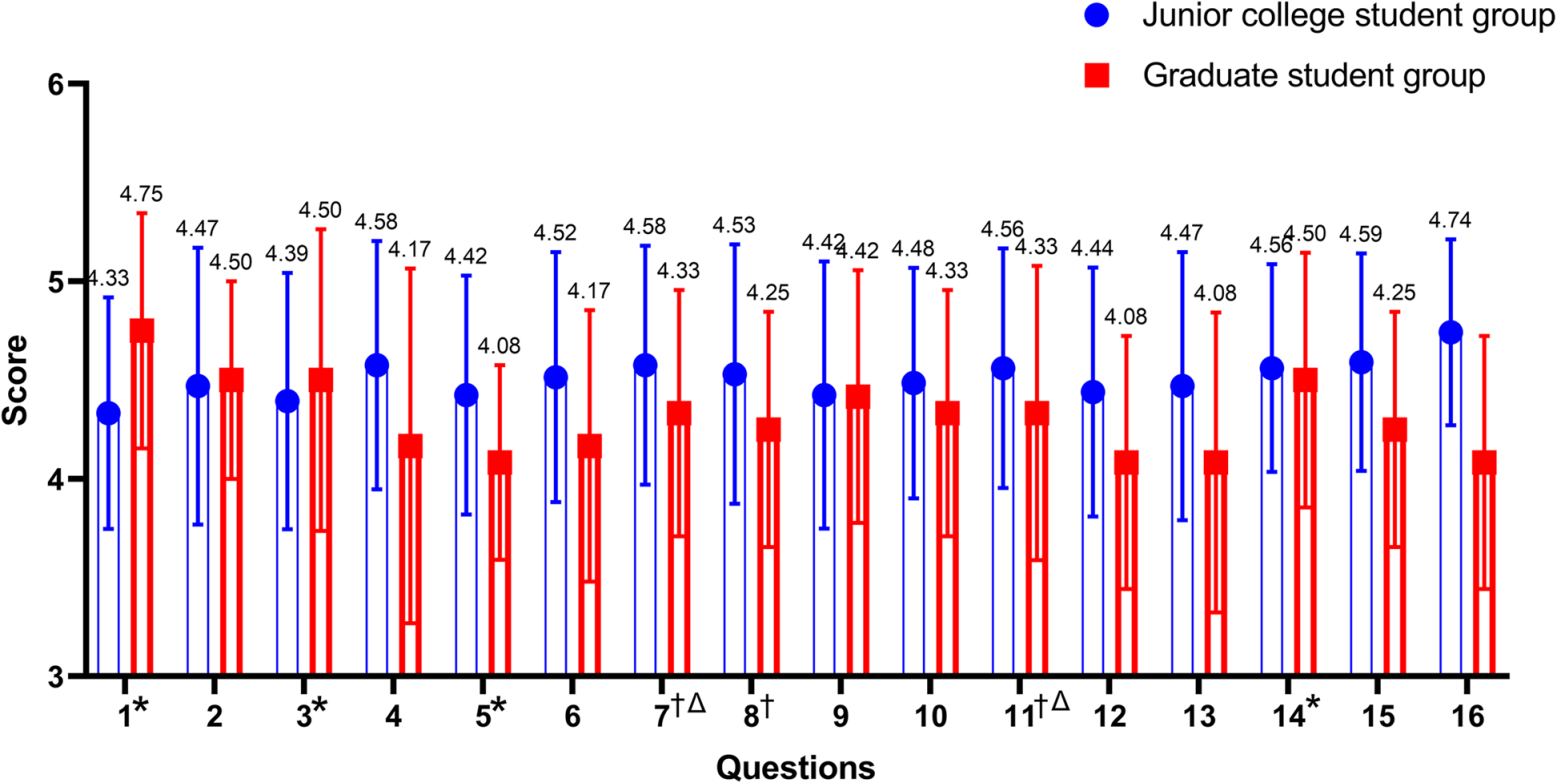
The mean score and standard deviation of groups of 16 questions in the questionnaire; *, *p* < 0.05 for comparison between groups; †, *p* < 0.05 for comparison with cricothyroid membrane puncture in question 5;△,*p* < 0.05 for comparison with pneumothorax puncture for airway obstruction in question 12

The medical students had high software satisfaction (Table 2 and Fig. 5, Q16, score: 4.64 ± 0.56). In the software interface aspect, the medical students thought that it was user-friendly and easy to operate (Table 2 and Fig. 5, Q1 and Q2). The average scores were 4.40 ± 0.61 and 4.49 ± 0.68, respectively. The results indicated that the Graduate student group more easily grasp the operation skills than the Junior college student group (the scores were 4.33 ± 0.59, and 4.75 ± 0.60, respectively, *p* = 0.041). In terms of the accuracy of 3D model structure and hemostatic puncture treatment (Table 2 and Fig. 5, Q3 and Q4), the software was considered accurate, with average scores of 4.41 ± 0.67 and 4.53 ± 0.69, respectively. The Graduate student group had significant differences with the Junior college student group in the accuracy of 3D model structures (the scores were 4.39 ± 0.65, and 4.50 ± 0.76, respectively, *p* = 0.042). Meanwhile, medical students thought that the software promoted a subjective learning initiative (score: 4.55 ± 0.549) and increased learning interest (score: 4.54 ± 0.57) (Table 2 and Fig. 5, Q14-15). The Junior college student group also thought that the software was more useful in promoting their subjective learning initiative (the scores were 4.56 ± 0.53, and 4.50 ± 0.65, respectively, *p* = 0.002).

**Table 2:** Teaching satisfaction questionnaire for the simulation software of hemostasis and puncture on the battlefield (Mean ± SD)

Number	Survey question	Junior college student group	Graduate student group	All students
1*	This system is easy to operate and learn, and I can quickly master its usage	4.33 ± 0.59	4.75 ± 0.60	4.40 ± 0.608
2	The interface of this system is attractive and user-friendly	4.47 ± 0.70	4.50 ± 0.50	4.49 ± 0.675
3*	The information on the three-dimensional morphology and adjacent relationship of the related anatomical structures is accurate in this system	4.39 ± 0.65	4.50 ± 0.76	4.41 ± 0.669
4	The operation information on pressure hemostasis and puncture is accurate in this system	4.58 ± 0.63	4.17 ± 0.90	4.53 ± 0.693
5*	This system can help me quickly master the knowledge of the applied anatomy and operation related to cricothyroid membrane puncture	4.42 ± 0.60	4.08 ± 0.49	4.39 ± 0.606
6	This system can help me quickly master the knowledge of the applied anatomy and operation related to finger pressure hemostasis for lower limb hemorrhages	4.52 ± 0.63	4.17 ± 0.69	4.46 ± 0.655
7†△	This system can help me quickly master the knowledge of the applied anatomy and operation related to finger pressure hemostasis for shoulder and arm hemorrhages	4.58 ± 0.60	4.33 ± 0.62	4.54 ± 0.615
8†	This system can help me quickly master the knowledge of the applied anatomy and operation related to finger pressure hemostasis for forearm or upper forearm hemorrhages	4.53 ± 0.66	4.25 ± 0.60	4.50 ± 0.656
9	This system can help me quickly master the knowledge of the applied anatomy and operation related to finger pressure hemostasis for forehead and neck hemorrhages	4.42 ± 0.68	4.42 ± 0.64	4.44 ± 0.672
10	This system can help me quickly master the knowledge of the applied anatomy and operation related to finger pressure hemostasis for facial bleeding	4.48 ± 0.58	4.33 ± 0.62	4.47 ± 0.595
11†△	This system can help me quickly master the knowledge of the applied anatomy and operation related to finger pressure hemostasis for bleeding of the vertex	4.56 ± 0.61	4.33 ± 0.75	4.54 ± 0.635
12	This system can help me quickly master the knowledge of the applied anatomy and operation related to pneumothorax puncture for airway obstruction	4.44 ± 0.63	4.08 ± 0.64	4.40 ± 0.648
13	This system can help me quickly master the knowledge of the applied anatomy and operation related to bone marrow puncture in hemorrhagic shock	4.47 ± 0.68	4.08 ± 0.76	4.41 ± 0.706
14*	This system can effectively improve my learning initiative	4.56 ± 0.53	4.50 ± 0.65	4.55 ± 0.549
15	This system can effectively improve my learning interest	4.59 ± 0.55	4.25 ± 0.60	4.54 ± 0.572
16	My satisfaction with this system:	4.74 ± 0.47	4.08 ± 0.64	4.64 ± 0.557
17	What are the advantages and disadvantages of this system compared with the training you have received before?	-	-	-
18	My comments and suggestions about this system:	-	-	-

*Note:* Arabic numbers 5, 4, 3, 2, and 1 represent strongly agree, agree, neutral, disagree, and strongly disagree, respectively; *SD,* Standard Deviation

^*^p < 0.05 for comparison between groups;

^†^*p* < 0.05 for comparison with cricothyroid membrane puncture in question 5

△*p* < 0.05 for comparison with pneumothorax puncture for airway obstruction in question 12

In terms of software application, all medical students agreed that the software was helpful for quickly mastering applied anatomy and operative skills on hemostasis and puncture treatment on the battlefield. The average scores of each injury modules were higher than 4.40 (Table 2 and Fig. 5, Q5-Q13), except for the cricothyroid membrane puncture module, which had the lowest score (score: 4.39 ± 0.606). The cricothyroid membrane puncture module score was significantly different from the finger pressure hemostasis for shoulder and arm hemorrhages, forearm or upper forearm hemorrhages, and bleeding of the vertex modules(*p* = 0.009, *p* = 0.046, and *p* = 0.000, respectively). Moreover, the pneumothorax puncture for airway obstruction module score was 4.40 ± 0.648, which was also significantly different from the finger pressure hemostasis for shoulder and arm hemorrhages, and bleeding vertex modules (*p* = 0.023, *p* = 0.004, respectively).

The comments in free text items showed that 78% said that the software is clearer, more intuitive, and helpful for learning. Regarding the deficiencies, the participants proposed that the bleeding effect should be added in the 3D interactive area to simulate arterial bleeding, which would permit a more realistic simulation effect. Adding combat application tourniquets, dressings, and fixation after first aid management were also suggested (Table 2 and Fig. 5, Q17-18).

## Discussion

First aid on the battlefield is essential to improve combat effectiveness during the ‘platinum ten minutes’ [8]. The “Buddy-Buddy” care system of the British Armed Forces emphasizes providing immediate assistance (including the use of hemostatic bandages and tourniquets) to wounded colleagues in combat units [3]. The Echelon I level of military medical care, which includes self and mutual rescue, was proposed by the U.S. Army [31]. The Tactical Combat Casualty Care (TCCC) guidelines of the U.S. Army simplify battlefield care into three phases. Here the use of tourniquets for controlling bleeding was placed in the first phase, named “Care Under Fire” [31]. Related reports have also shown that combat casualties treated with emergency tourniquets have a higher survival and lower incidence rates of blood loss [32]. However, Tsur [33] pointed out that conventional tourniquet training did not improve the success of tourniquet use by nonmedical personnel, accounting for 62.3% failure. The lack of comprehension, flawed basic skills, and skill acquisition might affect training under combat pressure. Therefore, it is crucial to carry out adequate self and mutual rescue training to correctly understand hemostasis compression points.

In this study, a treatment software was constructed based on the CVH dataset. It used 3D visualization and simulation technology to show medical students the 3D morphology and spatial relationship of injuries, and provided better study tools for simulating the treatment process effectively. Compared with traditional training modes, such as a medical simulation dummy [15], fake wounds, animal experiments, and pure theoretical study [16], our software could reflect true injury information and its detailed sectional and 3D topographic anatomy. This could help users learn and master first aid. Beaven [34] also reported a measurable increase in confidence for both technical skills in all major body areas, and non-technical skills upon using highly realistic trauma cadavers. However, he only provided real-world simulations of resuscitation and the physiological condition of the patient. His model lacked the simulation of wounds and the comparative study of anatomical knowledge, which is more suitable for students with rich medical knowledge.

After participating in the training, the medical students thought that our software provided good human–computer interaction and accurate 3D model structures. A strong knowledge of hemostatic puncture treatment could help medical students quickly grasp first aid techniques, including first-aid skills about hemostasis, pneumothorax puncture, and cricothyroid membrane puncture. However, they also thought that pneumothorax puncture for airway obstruction and cricothyroid membrane puncture were more difficult, which is consistent with previous studies [35]. It was mainly caused by the more complicated anatomy and high technical difficulty. We also found that the software is easier to operate for the graduate student group who noted that 3D anatomy structure was more accurate. It was mainly because the graduate students have a better clinical foundation, such that using the software and understanding the 3D adjacent structures were easier. Moreover, our results showed that the software helps motivate junior college students to learn. We attribute this to the sense of learning in games provided by our software.

The software can help medical students learn and understand anatomical knowledge related to first aid techniques more effectively and improve injury judgment accuracy and treatment proficiency. At the same time, war trauma has common features with traffic and disaster injuries, e.g. instantaneity, mass, and frequency characteristics, and most traffic and disaster injuries are penetrating, blunt, blast, and concussion injuries. Tannvik [36, 37] et al. suggested that bystanders with first aid training experience could provide better first aid than those with unknown first aid training. Therefore, the first aid training for war trauma constructed in this study comprises extensive knowledge on first aid techniques. This can also be used for providing first aid training for traffic and disaster injuries.

## Limitations

In this study, the simulation software demonstrated the war trauma effects from three- and two-dimensional perspectives and provided a comparison display with cross-sectional anatomical images. However, this study has limitations: first, more methods, including experiments or focus groups, should be used to verify the training effects of our software. Second, the software should be used to teach soldiers self and mutual rescue. Moreover, the number of participants should be further increased. Third, simulation fidelity and content of our software should be improved using virtual reality, augmented reality, and mixed reality, as well as other technologies. For example, adding bleeding animations, optimizing the pressure point radius range, including different pressure areas to suggest other treatment effects, and providing appropriate supplementation of treatment courses following emergency hemostasis (e.g., courses on bandages, tourniquets, and fixation). Fourth, the software lacked real mechanical feedback that could be effectively remedied by learning from a real human body. Therefore, various training modes, such as medical training mannequins and fake wounds, should be integrated into the software in the subsequent iterations of the software. Finally, the software is currently a preliminary version, and we will make further improvements based on training comments, including developing an English version.

## Conclusion

The war trauma treatment simulation software, which was created based on a high-resolution, thin-sectional and true-color anatomical CVH dataset, has attractive and user-friendly interface. This can provide detailed 3D anatomical morphology and adjacent relationship, and can help medical students to quickly master the knowledge of the first aid treatment skills and its applied anatomy. This is also suitable for emergency self-rescue in traffic and explosion injuries, and can provide officers and soldiers with first aid knowledge. 

## Supplementary Information

**Additional file 1:** (AVI 338197 kb)

**Additional file 2:** **Fig.S1** Software frame diagram of the treatment simulation software on the battlefield a, First-level interface; b, Second-level interface; c, Third-level interface; d, Fourth-level interface. **Fig.S2** Learning module of pressure hemostasis of the foot a, Injury demonstration. b. Error message when pressed wrong position; c, Demonstration of the correct hemostatic pressure point after transparency of the skin; d, Operation of the correct pressure position and confirmation to proceed to the next step; e, Animated demonstration of pressure position; f, Teaching video of pressure techniques. 

## Data Availability

The datasets analyzed during the current study are available from the corresponding author on reasonable request.

## Abbreviations

CVH: Chinese Visible Human
3D: Three-dimensional
