# Supplementary material for: Creation and application of war trauma treatment simulation software for first aid on the battlefield based on undeformed high-resolution sectional anatomical image (Chinese Visible Human dataset)
Source: BMC Med Educ. 2022 Jun 26;22:498. doi: 10.1186/s12909-022-03566-6 (PMC9233836; doi:10.1186/s12909-022-03566-6)
Supplement: Supplementary file 2 — Additional file 2: Fig.S1 Software frame diagram of the treatment simulation software on the battlefield a, First-level interface; b, Second-level interface; c, Third-level interface; d, Fourth-level interface. Fig.S2 Learning module of pressure hemostasis of the foot a, Injury demonstration. b. Error message when pressed wrong position; c, Demonstration of the correct hemostatic pressure point after transparency of the skin; d, Operation of the correct pressure position and confirmation to proceed to the next step; e, Animated demonstration of pressure position; f, Teaching video of pressure techniques. [file 12909_2022_3566_MOESM2_ESM.docx]

Supplementary materials


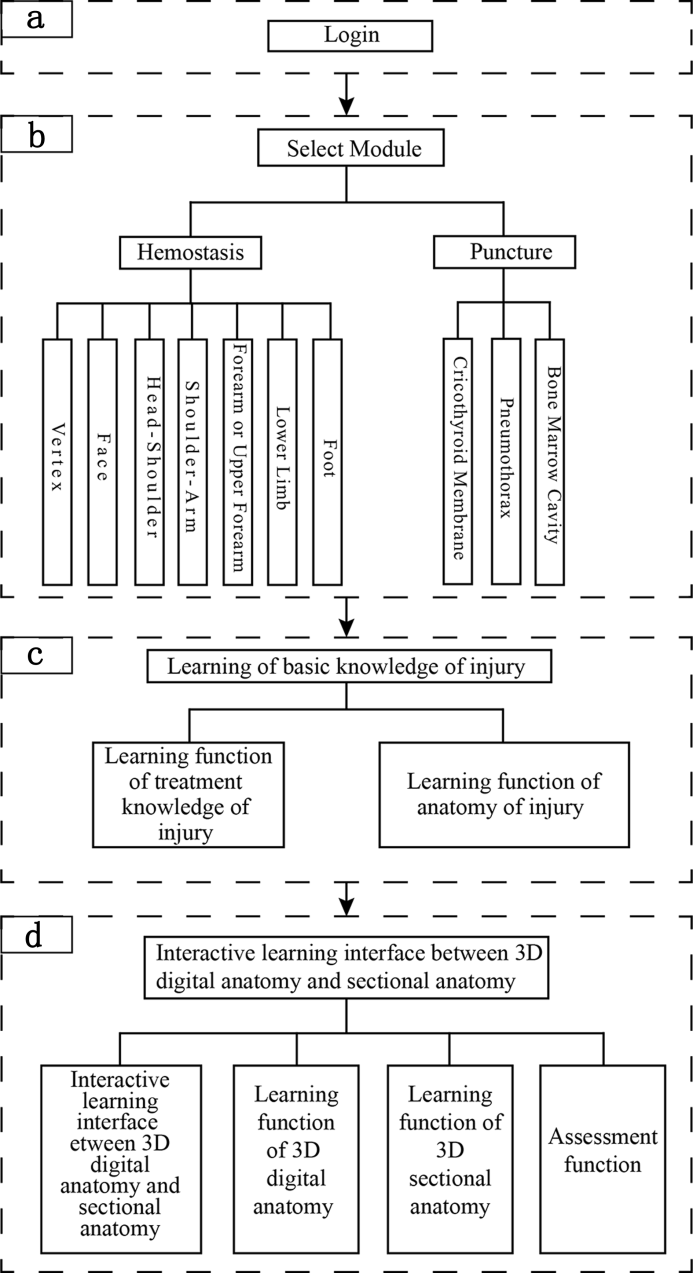


**Fig.S1** Software frame diagram of the treatment simulation software on the battlefield a, First-level interface; b, Second-level interface; c, Third-level interface; d, Fourth-level interface.


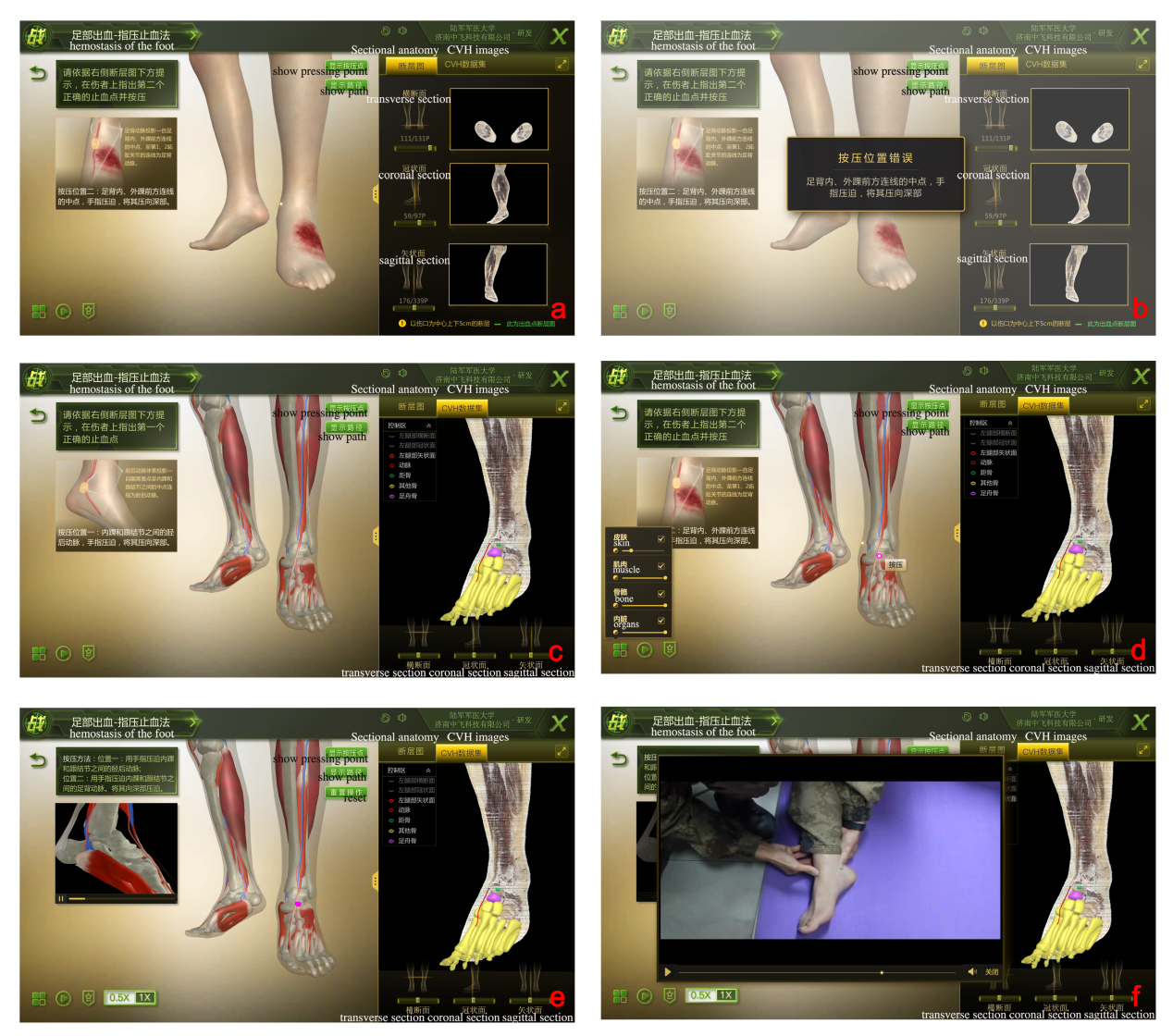


**Fig.S2** Learning module of pressure hemostasis of the foot a, Injury demonstration. b. Error message when pressed wrong position; c, Demonstration of the correct hemostatic pressure point after transparency of the skin; d, Operation of the correct pressure position and confirmation to proceed to the next step; e, Animated demonstration of pressure position; f, Teaching video of pressure techniques.
